# Obstetrics

**Published:** 1857-05

**Authors:** 


### OBSTETRICS.

1. ON THE USE OF ICE IN UTERINE HEMORRHAGES. By *E. A. Hildreth*, M.D., Wheeling, Va.—[The introduction of ice into the cavity of the uterus, although a very old remedy, is one which has never been much used in this country, especially in country practice, and it is only for the purpose of reminding practitioners of its potency and efficacy in uterine hemorrhages, that we copy from the February No. of the Cincinnati Medical Observer, the following report of four cases, thus treated.] "The safety of passing a quantity of ice into the cavity of the uterus after the expulsion of the child or placenta, has been questioned by some, as, I believe, on purely theoretical grounds. The effect in every case I have used it, has been to contract the uterus quickly, energetically, and permanently; and as a matter of course stop the uterine flow. I have yet to see any unpleasant result, directly or indirectly arising therefrom; on the contrary, the relief afforded has been prompt and permanent.

"I do not wish to theorize on the subject at this time. Allow me to subjoin a few *facts* as observed and noted when they occurred.

"*Case* I. June 16th, 1846, Mrs. McC., æt. 40, in labor with her fourth child. Describes her previous labors as 'lingering.' On examination by the touch found the os uteri thick, firm, opened as large as a half dollar; membranes entire; breech presenting; pains slight. Prescribed pulv. ipec. comp. grs. xii, and left, requesting them to call on me when the pains became more active. Called back in 6 hours, found her pains strong and expulsive, and half an hour after the child was expelled. Upon introducing my hand along the umbilical cord, it was ascertained that hour-glass contraction was present; the placenta remaining at the fundus of the uterus. An attempt was made slowly to pass the hand through the contracted portion, but failed. Gave her morphia sulph. grs. ss, and permitted her to rest. Says she has felt no pain since the birth of the child. In about half an hour she had some pain, with profuse hemorrhage. Used affusion of cold water over abdomen with pressure, and gave her, ℞. Morphia acet. grs. ¼, plumb. acet. grs. iii. Flooding is checked. Endeavored to extract placenta, but failed. In about fifteen minutes flooding returned to an alarming degree. Placed pounded ice over hypogastrium, and introduced several pieces into the vagina as high as os uteri; flooding restrained and hour-glass contraction relaxed, so that the hand could be introduced and placenta extracted. Five minutes after the flooding returned. Passed my hand into the uterus in hope of provoking contractions, but without effect—it feels like a *wet leather bag*. Pulse very small and frequent, face and lips pallid; complains of faintness and dizziness. Fearing now her rapid dissolution unless a more successful treatment was pursued, I seized a lump of ice as large as a lemon, and carrying it through the os uteri slipped it from my hand. The effect was immediate and powerful, expelling a quantity of coagula, and contracting the uterus to its usual size and firmness; a graduated compress and bandage were then applied; pulse 120, small, weak; complains of giddiness. M. M. perfect rest in horizontal posture; pulverized opii. grs. iii, immediately after rest, panada with brandy. Saw her four hours after; has slept some; no return of 'wasting;' feels comfortable; pulse 100, soft, full; womb well contracted; no pain on pressure.

"17th. Feels well; slept well last night; pulse 90, weak; likes her panada; no pain or tenderness over abdomen; lochia not more free than usual; M. M. let her rest.

"18th. Doing well. 22d, thinks she can sit up; forbid it, and discharged her, well.

"*Case* II. Nov. 7th, 1847. Called in haste to see Mrs. B.; found a German woman attending her as midwife; the child had been born about an hour; placenta not delivered, but she was flooding profusely, which alarmed the midwife; removed the placenta and gave morphia grs. ss; friction with pressure over uterus; sent for ice; hemorrhage somewhat less; her face is blanched and anxious; pulse very frequent; passed a lump of ice as large as a walnut *into the uterus*, which was followed by expulsion of coagula and firm contraction; repeated morphia gr. ¼, and left her in care of midwife. She had no return of hemorrhage, and subsequently did well.

"*Case*. III. Called in consultation with Dr. W., of M., to see Mrs. M., a large, fleshy woman, who had had miscarriage at the third month of utero-gestation. The fœtus had been thrown off for several hours, but placenta retained; frightful hemorrhage supervened, during which she had twice fainted while in the horizontal posture; the placenta could be felt through the os uteri with the point of the fore-finger; sent for ice, and during the absence of the messenger, endeavored to extract the placenta with Dewees' placental hook, but failed. Dr. W.

had previously used morphia, acetate of lead, cold applications, etc., but the flooding continued, though in a more moderate degree. From her general appearance, coldness of surface, feeble pulse, etc., concluded she must soon sink if not quickly relieved. At this juncture the ice came, and we prepared a crystal about the size of the index finger, and passed it through the os into the cavity of the uterus, as far as possible, and allowed it to melt; the flooding ceased and did not return again, although the placenta was not thrown off for 30 hours afterward. She recovered.

"*Case* IV. April 22d, 1849. Mrs. O., after a natural and easy labor, was delivered of a second child; placenta followed in 15 minutes; bandaged her and left her doing well.

"May 30th, called to her in haste; says she was taken 'unwell' yesterday; the discharge growing more profuse ever since (there is a case of cholera in the next room, and she is badly frightened); her bed is now saturated with blood, and she is flooding rapidly; os uteri easily admits the fore-finger, and is soft and dilatable. Gave her acet. plumbi and opium, applied douche of cold water, ordered ice and used the plug; hemorrhage still profuse; complains of giddiness and singing in the ears; pulse very frequent and feeble; face blanched; reapplied cold douche, but without effect. Her husband, after considerable delay, brought the ice; I removed the plug, which was followed by a considerable gush of blood; introduced into the uterus several pieces of ice the size of a chestnut, with the effect of stopping the flooding instantaneously. May 31st. Has had no recurrence of hemorrhage since the use of the ice. June 1st. The discharge from the uterus scarcely stains her cloth. She recovered.

"We hope the above detail is sufficient to give an idea of its application. We have never tried it in a case of placenta previa. As to its being 'something new,' I do not know nor care; if by making the practice more generally known through the pages of your valuable journal, I become instrumental in saving one poor woman from death by uterine hemorrhage, I shall be fully compensated."—*Medical Observer*, February.

2. A CASE PROVING THAT MENSTRUATION IS NOT ONLY OVULATION, WITH OR WITHOUT A SANGUINEOUS DISCHARGE; BUT THAT IT IS ALSO THE PERIODICAL EXFOLIATION OF THE MUCOUS MEMBRANE OF THE BODY OF THE UTERUS. By *D. W. Brickell*, M.D., Prof. Obstet. N. O. School of Med.—"About six months ago, I was performing an autopsy in the dead-house of the Charity Hospital. On an adjoining table lay the body of a stout young female, who was said to have died of disease of the heart. She had died a few hours previously, and was still quite warm. The thorax and abdomen were laid open. The body had been abandoned, and curiosity led me to examine the internal organs of generation. The uterus and appendages had been cut from the pelvis, and the anterior wall of the organ had been laid open. The moment I saw the organs, I was struck with their being highly engorged with blood, and the uterus was considerably larger than usual. The pelvis was filled with blood, which had flowed from the vessels when the organs were detached. The next thing that attracted my attention was the most palpable specimen of *recent* corpus luteum in one ovary. The corpus was large and prominent, and the depression on its centre, exhibiting the point of escape of the ovule, was evident beyond all cavil. In this same ovary one other Graafian vesicle seemed fully matured, the parts surrounding it being highly congested, but the ovule had not escaped. The other ovary was generally congested, but there appeared to be no mature Graafian vesicle.

"But the most interesting feature in the case, was the *complete absence of the lining membrane of the cavity of the body of the uterus.* The moment my eye alighted on the inner surface of the organ, I recognized the woodcut of Tyler Smith, in the May, 1856, number of the *Lancet* (Amer. edition), representing the inner surface of the uterus of a woman who died of apoplexy during the catamenial flow. Nothing could have been more striking than this resemblance; and if I had ever been sceptical in relation to the observations of the author, I was now bound to admit his accuracy. Down *to the os uteri internum* the mucous membrane was gone, and the inner surface of the organ rough, with innumerable blood-spots scattered over it. All *below* the os internum was smooth, and in every respect natural in appearance. The difference in sensation conveyed to the finger by touching the two surfaces was as palpable as the impression conveyed to the eye.

"The only doubt now remaining about the case was, whether it might not be a uterus which had very recently been delivered of an early ovum. More extended examination, however, proved clearly that this was not the case. The vagina was very small, and its mucous membrane highly corrugated; and there was a well-defined hymen. To add to this the mammæ showed none of the changes generally produced by early pregnancy.

"The subject was, to all appearance, about eighteen or twenty years of age, and quite robust. She was the subject of anasarca to a considerable extent, and was said to have died very sudcenly—her death being attributed to disease of the heart. I tried to get a more accurate history of her from the nurse of the ward in which she died, but, as is too often the case, she only knew that such a woman had been in the ward, had lived, and then had died.

"Tyler Smith says, 'According to my view, the mucous membrane of the uterus becomes excrementitious every month, and is discharged from the cavity of the uterus in a state of disintegration, and the uterus forms a new mucous coat, by a process similar to the reproduction of lost parts.' Coste and others speak of the exfoliation of the mucous membrane of the uterine cavity under certain circumstances; but, so far as I am aware, Tyler Smith is the original advocate of the theory above laid down. After reading all the observations I could procure on this interesting subject, I was altogether inclined to adopt this theory, and the case I have thus described only the more strongly tends to prove its correctness."—*N. O. Med. News and Hosp. Gazette,* Feb. 1857.

3. HEMORRHAGE AS A SIGN OF CANCER OF THE UTERUS.—" Dr. West remarked, in his out-patient's room, at St. Bartholomew's, on the almost constant occurrence of hemorrhage as a symptom of commencing cancer of the os uteri. He believed, he said, that it was quite as constant and valuable a sign, in relation to that disease, as hæmoptysis is in respect to tubercle in the lungs. Of course, inasmuch as the uterus is in health subject to sanguineous discharges, there is need of care in determining that the sign be really one of disease; that, for instance, it occurs with an irregularity, and a profuseness greater than disturbed catamenial function could account for. The symptom has its peculiar value when the subject of the affection has previously ceased to menstruate. Dr. West stated, that he had long recognized the importance of the symptom, but that on recently counting up his cases of uterine cancer he had been astonished to find how almost invariably it had been the earliest sign of the existence of the disease."—*Times and Gazette*, Feb. 21, 1857.

4. STERILITY DEPENDENT ON DISEASES OF THE RECTUM. By *I. Baker Brown*, F.R.C.S.—"I shall now proceed to speak more fully of a series of causes of sterility, which, as before said, have not been previously recognized, viz., diseases of the rectum. Let me first recall to your minds the general law of the animal economy, 'That any irregularity or interference with the functional action of any one part of the body affects more or less the whole body.' If this law pertain to the body generally, how much more must it obtain between the female organs of generation, where the slightest deviation from normal functional action must materially interfere with the delicate physiological process of impregnation, and the contiguous organs. We must bear in mind that both the rectum and uterus are supplied with blood from the internal iliac artery, and with nervous influence from the sacral plexus, and that, therefore, disease or functional derangement in the one part or organ must interfere with the other. This I desire to illustrate in the following manner: A female is suffering from bleeding hemorrhoids. At the menstrual epoch there is an increased supply to the hemorrhoidal vessels; and consequently, a diminished supply to the uterus, because nature only sends down a sufficient supply for the uterine function. The mucous membrane of the uterus will not, therefore, in this case undergo those normal changes which are necessary for the reception of the impregnated ovum. The same observations apply to prolapsus ani, where there is always some loss of blood at every time of defecation, and a greater loss at the period of the menstrual epoch. I could illustrate this with many cases, but one will suffice:

"*Sterility from Prolapsus Ani.*—Mrs. H., aged 33, married ten years, without family, consulted me on account of prolapsus of the bowel at every act of defecation, accompanied by loss of blood. The general health had greatly suffered. Upon questioning her, she confessed that during married life the catamenia had been scanty, thin, and light in color; but that during the menstrual period much blood was lost from the bowel. The affection of the bowel, I pointed out, was readily curable; and, as there was no uterine disease to be detected, that the state of the bowel was the probable cause of her having had no family.

"After preparatory regimen and medicine I proceeded to operate a week after the menstrual epoch. The bowels having been previously freely opened. I applied three separate ligatures to the prolapsed mucous membrane, and returned the tied parts within the sphincter. Opiates were freely given, and continued for a week so as to keep the bowels confined, and she was placed on generous diet, with wine. This patient progressed most favorably, the bowel was completely relieved, and at the next catamenial period, the uterine discharge was much augmented both in quantity and color. Tonics and good diet were persevered with, and she lived apart from her husband for two months. Soon after this period she became pregnant, and advanced to the full period, and was delivered of a fine healthy child.

"If a patient is suffering from fistula or fissure, there is constantly more or less pain in the uterus as a result of reflex action; and, consequently, it is always under a state of irritation, which renders it unfit for the quiet and perfect performance of its duties. Indeed, I have seen many cases, which I shall mention on a future occasion, of patients having been treated for months and years for uterine inflammation with leeches, caustics, &c., where I have discovered a long-standing fissure of the bowel, which has been the sole exciting cause of the uterine affection. I shall here give a case.

"*Fissure of the Rectum.*—Mrs. M., aged 26, married three years, without children, consulted me and made the following statement. Ever since her marriage, she had suffered from more or less pain about the womb, not acute, but of a dull, wearing kind. Catamenia were regular, both as to time and quantity, and she suffered very slightly from leucorrhœa; had no pain on sexual intercourse, but felt generally ill, and suffered from dyspepsia. An examination per vaginam satisfied me there was no disease of the uterine organs. On further inquiry, she told me she suffered constant pain at stool, and frequently passed a small quantity of blood; a local examination showed me a deep and long-standing fissure of the rectum, which, to my mind, explained all her symptoms, and was the probable cause of her sterility. After preparatory treatment, I accordingly operated in the usual way by dividing the fissure. The after-treatment consisted in placing her under the operation of opium, giving her at once two grains, and every four hours after, one grain, for the first twenty-four hours, then for the next day one grain every six hours, and for the next week a grain night and morning; my object and principle being not only to secure perfect quiet for the bowel, but to obviate all pain. With this treatment was conjoined generous diet, and, according to rule, the recumbent posture was insisted on, until the parts were well healed; at the end of nine days the bowels were opened by repeated injections of tepid water. This plan of confining the bowels after operations about the rectum and perinæum, the use of generous diet and wine, and the subsequent administration of enemata, has received a full consideration, both in some papers I have had the honor of reading before the Medical Society, and in my work on the treatment of some important surgical diseases of women.

"From the foregoing observations it will readily be seen that ascarides in the rectum, or any irregularity or disease of that bowel, must necessarily be a frequent cause of sterility. It is very gratifying, however, to know that when once the mind has been drawn to this fact, the cases become more simple and among the most readily cured."—*Times and Gazette*, Feb. 21, 1857.

5. DISCUSSION AT THE PARIS ACADEMY OF MEDICINE UPON THE TREATMENT OF OVARIAN CYSTS.—This discussion, which had been continued over many meetings, has recently terminated, and a concise summary of the opinions advanced will no doubt be of some interest to our readers. They will learn with some surprise, that the operation of ovariotomy, under any circumstances, is absolutely ignored by the French surgeons; and the cases in which neither the palliative treatment, nor the treatment by iodine is admissible, are quietly left without any attempt being made to avert certain death. A shuddering horror is expressed at the rash conduct of British and American practitioners, as if none but the most prudential surgery ever prevailed on the other side of the Channel; disbelief is expressed at the amount of success recorded, while the mistakes in diagnosis, which it must be acknowledged have been of too frequent occurrence, have evidently made an impression greater than is justifiable.

The discussion was occasioned by the relation of a case by Dr. Barth. The patient, 37 years of age, had suffered from ovarian disease during two years, when, on the 10th of March, two punctures were made, in such a way that a gum-elastic tube, pierced with holes, was passed in at one end and out at the other, so as to allow of the tumor being retained against the abdominal wall, while its contents could be discharged and injections thrown in. We may mention, *en passant*, that this mode of procedure, or that of leaving a canula in the opening, was generally disapproved of by the Academy. Ten days afterwards an iodine injection was thrown in, and repeated for some days; the tumor diminished in size, but the patient getting tired, left the hospital. On the 26th of May, however, she was brought back in the pains of childbirth, although she had been quite unaware of her pregnancy, and was delivered the same day of a five months' child. Acute peritonitis followed, and she died on the 28th. At the autopsy, the cyst was found half filled with the same albumino-purulent fluid that had flowed through the canula during life, while at its upper part was a round opening the size of a sixpence, through which some of the fluid had gained access to the peritoneum. Behind the tumor, the uterus had contracted, and presented the appearances usual after delivery.

M. Malgaigne commenced the discussion by raising the question, whether these tumors should be let alone or treated by palliative punctures. Such punctures are, as a general rule, inoffensive, and give relief; but while women are found coming to the *Bureau Central*, undergoing the puncture, and walking home again (!), others of them die within the twenty-four hours. When danger of suffocation exists, however, puncture is evidently called for. As to the curative powers of iodine injections, he believes the reputed cases require verification; while, if they have really taken place, nature may have effected them in spite of the surgeon. Some amount of contraction of the cyst may be determined by the iodine; but, as this is only limited, a dangerous fistulous opening may be left. As at present advised, he does not go beyond mere palliative puncture, and that only in certain cases.

M. Moreau observed, that the coincidence of these cysts with pregnancy is by no means rare; several of such cases having come under his notice, which, while the cysts were large enough to require puncture on account of the obstacle they placed to delivery, yet had they not interfered with the progress of pregnancy, which reached its full time. He believes the best plan is to meddle with ovarian tumors as little as possible, for some patients have these cysts for fifteen or twenty years without their health being inconvenienced. When they make considerable progress, palliative punctures are alone called for. In one of M. Moreau's patients these were performed twenty-eight times in twenty-eight years, and in another one hundred and ten times.

M. Cazeaux has paid much attention to this subject, and he is certain that cures may be obtained by means of iodine injections; for he has seen some of M. Boinet's patients three and five years after, when all that remained was a solid tumor, about the size of the fœtal head, possessing none of the characters of cysts. The treatment is not applicable to multilocular cysts, or when there are malignant complications, and when from faulty diagnosis, it is resorted to in such cases, death may be the result. Ovarian cysts are by no means so inoffensive as stated by M. Moreau, and the retention of health in spite of their presence is quite exceptional. Tapping generally becomes indispensable; and after this has been repeatedly performed a fatal result usually takes place at the end of a few years. M. Cazeaux also regards the complication of pregnancy by these cysts as a much more serious occurrence than M. Moreau represents it.

M. Velpeau believes that, perhaps, in the majority of cases, women having these tumors, may enjoy tolerable health for ten, twenty, or thirty years; in fact, the mean of ordinary life: but still there are others not so fortunate, but who succumb within the first ten years, whether from the excessive development of the cyst, its inflammation, the tapping it has rendered necessary, or intercurrent diseases. As to the palliative tapping, M. Velpeau had performed it so often that he had come to look upon it as a very simple affair, when he lost several patients in the course of a single year. As, for a reasonable chance of effecting a radical cure in these cases, the woman's health must be tolerably good, the surgeon is placed in the dilemma of determining whether he shall propose to a woman in good health an operation which may kill her, or abandon her to a disease that may become dangerous. Great as has been M. Velpeau's employment of iodine injections in other circumstances, he has always felt reserve in using them in ovarian cysts. Numerous facts have shown him that they exert remarkable power in dropsies of serous membranes, and that their success is less in proportion as the surface to be modified differs in structure from these. In cysts containing uncoagulated blood they may still exert good service; but they are of less use when the contents are gelatiniform, still less when puriform, and scarcely of any utility when the parietes resemble mucous membrane in structure. Observing the success of others, M. Velpeau has latterly had recourse to these iodine injections himself, and with some good results; and as he believes the injection of the iodine does not add to the danger of tapping, he thinks there is no reason why an attempt at curative should not be substituted for mere palliative treatment.

M. Demarquay detailed to the Academy the result of several cases treated by himself and M. Monod. He believes that emptying these cysts all at once, when large, excites disturbance of the system, and presents too large a surface for the efficient action of the iodine. Availing himself of the shrinking which takes place in the cysts, he first only draws off one half of their contents; and then, a week after, when the walls of the abdomen and of the cyst have retracted, he removes two-thirds of what remains, and some days later completely empties the cysts by a third puncture, and throws the iodine into the now diminished cavity. A large trocar should be used, and the entrance of air guarded against.

M. Trousseau.—In the majority of cases ovarian cysts constitute neither a disease nor an inconvenience; that is to say, when their dimensions are small, as that of an orange or less, in which state they are often met with in the bodies of the aged. The bursting of simple serous cysts also gives rise to no ill effects. M. Trousseau never met with a case of their spontaneous absorption, unaccompanied by inflammatory action. We must always bear in mind that these tumors, like fibrous bodies of the uterus, may, under the influence of the menstrual *molimen*, acquire a great size, and then diminish again; and the diminution that has taken place in some cases, during the employment of iodine, may in this way be explained. The results of palliative tapping would, probably, be more fortunate, if it were practised when the cyst is small, *i. e.*, not exceeding a child's head in size; for, in the case of inflammation supervening upon the operation, this is less severe in proportion to the smaller size of the cyst, while the walls are more retractile, not having yet assumed the morbid conditions they will do at a later period. As to iodine injections, feeling somewhat afraid of them, M. Trousseau has not yet sanctioned their employment in any of his patients, and he questions the durability of the cures said to have been obtained by others.

M. Jobert observed, that although simple tapping is regarded by some as an operation devoid of danger, under certain circumstances it is a very dangerous one. A capital distinction should be made between *adherent* and *movable* cysts, the operation never in the former case being attended with danger, while in recent, free, floating cysts, the fluid may get into the cavity of the peritoneum, and give rise to fatal diffused peritonitis. To prevent this, the canula should be maintained *in situ*, in order to determine adhesive inflammation between the contiguous surfaces of the cyst and the abdominal wall. No accidents have ever followed M. Jobert's operations when thus conducted. He has never observed a cure to result from simple tapping; but in two cases it has done so after multiple punctures, which have been followed by the deposition of plastic lymph in the substance of the tumor, and the obliteration of the sac. He has employed the iodine injections in thirty cases, and has never, observing the above precautions, met with any serious accident. In several cases a relapse has occurred, although the tumor had seemed to have been completely obliterated. So far from exciting inflammation, the iodine arrests it when present; just as in the case of hydrocele, it at once moderates the symptoms of orchitis. The greatest analogy, in fact, prevails in the results obtained from its injection into the tunica vaginalis, and into these cysts. At first the fluid is reproduced, giving to the cyst its original size; but it then becomes condensed and concretes. M. Jobert regards these injections as inoffensive, providing that the inflammation by which they are followed does not extend over too large an extent. Evidently, a cure cannot always be looked for, *e. g.*, in cysts having cartilaginous walls, or containing malignant deposits, and multilocular cysts have been placed in the same category. In a case of multilocular cyst of the thyroid, however, M. Jobert dispersed the whole tumor by injecting one of its cells; and he has known a complete cure of a multilocular ovarian cyst take place after six applications of electricity. To sum up: palliative tapping is never dangerous when there are adhesions to the wall of the abdomen. Puncture, leaving a fistulous opening, is a dangerous proceeding. Iodine injections and electricity, exempt from serious inconveniences when carefully employed, may lead to a cure, without our being as yet able to explain the mechanism of their operation.

M. Piorry observed, that when the walls of the cyst are thin the chances of success with the iodine are much greater, while they are very slight when these have become thickened and firm, and formed of different tissues, or of fibrinous, or even cretaceous substances. Very large cysts, which induce dangerous symptoms by their compression, must be tapped, whether adherent to the walls of the abdomen or not; and such palliative tappings may prolong lives for many years that would otherwise have been lost by the progress of the disease. For a long time past M. Piorry has advocated, like M. Demarquay, the discharging a portion of the fluid first, and only injecting the iodine at a later period. When with a very large cyst, there exist also several small ones, iodine injections are indicated; and if two or three of large size are detected, these may be successfully attacked. The partitions of these cysts may also be perforated with the trocar, so as to discharge their contents at a single external opening; but when there are a great number of cystoid tumors united into a mass, palliative puncturing is alone indicated. When, independently of the large ovarian cyst, solid, irregular, adherent productions, coexist, little is to be hoped from the use of iodine; but while employing palliative puncture of the cyst, the solid tumors are sometimes favorably influenced by douches, iodine frictions, and in certain cases, even by a kind of shampooing and compression. Early tapping of an ovarian cyst is indicated, when, by its thrusting the viscera towards the thorax, it threatens suffocation; when the compression of the vena cava and vena porta induces ascites or œdema; when the passage of matters through the alimentary canal is obstructed, or when painful prolapsus of the uterus is induced. In general, too, an ovarian cyst, which by the abundance of fluid it gives rise to induces extreme hypoæmia, will be advantageously treated by its prompt evacuation and the throwing in of iodine. An ovarian cyst containing pus should be opened only after adhesions have been induced between it and the abdominal parietes. Then, its contents should be completely evacuated, either by puncture or large incisions, its interior washed out, and iodine injections thrown in daily until pus ceases to flow.

M. Piorry is of opinion that, whether large or small, the evacuation of ovarian cysts should be as complete as possible; and to secure the complete discharge of the fluid, whether in these cases or in ascites, he has long been in the habit of passing a gum-elastic catheter through the canula, and constituting it a syphon. He also attaches importance to the prevention of the penetration of air during the tapping, and this he does by applying over the point to be punctured a glass cylinder having no bottom, and filled with water. Puncturing through this, some water may enter but no air; and to the same end, the extremity of the syphon may be placed in water. He also recommends two other precautions. 1st. The tumor should be most carefully percussed, the shades of dulness observed, and their limits traced with a pencil. In this way we soon get to know whether there are more than one cyst, and the positions and relations of these; and, especially, whether there is a noose of intestines between the tumor and the wall of the abdomen. Without this precaution the intestine, supported by the tumor, is very likely to be pierced through and through. In ascites this does not take place, for the intestine being mobile, and unsupported behind, the trocar does not traverse it. M. Piorry has tried numerous experiments upon the dead body, and has usually found, when adhesions have not been present, and the intestines have only been distended by air, even the sharpest trocar will rather push them before it than penetrate them. 2d. The position given to the patient is of great importance. M. Piorry always punctures at the side, and, having completely emptied the cyst by means of a syphon, places the patient during the next twenty-four hours on the opposite side of the body. In this way no fluid can reach the peritoneum, and induce its inflammation.

M. Gimelle, although aware of exceptional instances of these tumors remaining stationary for twelve or twenty years, is an advocate for interference, when the disease continues to make progress so as to threaten to reach that large size, which, after a greater or less number of punctures, always terminates fatally. The conditions justifying iodine injections, in his opinion, are: 1st. When the cysts present no abnormal changes of structure, being neither cartilaginous nor indurated, and giving no sensation of hard or scirrhous bodies within. 2d. When its size does not exceed a diameter of ten or fifteen centimetres, or when it is larger, after it has been first reduced in size by frequent preliminary tappings; so that in the event of inflammation occurring, it may be limited to as small a space as possible. He then throws in thirty drachms of tincture of iodine, diluted with eight or ten of water, leaves it in for some minutes while exerting gentle pressure on the abdomen, then evacuates as much as possible without employing compression, and leaves the patient in a state of absolute repose. He cites two cases, occurring in young women, in which this practice was successful.

Dr. Schnepf communicated to the Academy the results of Dr. Fock's experience in the iodine treatment, which he has employed in fifteen cases. Of these, nine were radically cured after from one to six injections. Of the six others, in two cases a cure followed keeping the wound open and repeated washings out with iodine injections; in one, similarly treated, purulent infection followed. In two others, complicated with cancer, the iodine hastened the catastrophe, while in the last patient it exerted no effect.

M. Cruveilhier.—So parasitic is the life of ovarian cysts, that they remain completely strangers to all the great vital and organic movements of the economy; so that while dropsies of serous membranes may be often advantageously treated by internal medicines, these encysted dropsies are quite refractory. It is to surgical treatment that we can alone look with any hopes of success, but our decision to have recourse to this should be materially influenced by the anatomical characters of the cysts, which are far from being always the same. The differences are dependent upon the quality of the fluid, the disposition of the cyst, and its structure. (1.) It is of great importance, as regards facility of evacuation, whether the fluid be serous, viscous, albuminous, or gelatiniform, and the character of the fluctuation will to a certain extent enable us to pronounce upon the nature of this fluid. (2.) The cysts may be either unilocular, multilocular, areolar, or vesicular, and compound, the latter resulting from a union of the other varieties. (3.) In structure the unilocular cyst sometimes exactly resembles a normal fibro-serous sac, its interior being as smooth as if lined with serous membrane; but it is by no means rare to find the inner surface rugous, or raised by papillæ or vegetations of varying hardness, or occasionally the walls may contain cartilaginous or even osseous plates. In one of the varieties of unilocular cysts there are numerous imperfect divisions, allowing of intercommunication between the compartments. In what M. Cruveilheir terms areolar cysts, of which the vesicular is but a variety, the ovary has become transformed into an areolar mass, having communication between its meshes, and filled with albuminous matter, varying in consistency from the white of egg to that of honey or jelly. The viscous nature of its contents explains the difficulty or impossibility of evacuating it after tapping. These cases may be regarded as absolutely incurable, not from their nature, which is not malignant, though they greatly resemble in appearance gelatiniform and areolar cancer, but owing to the viscous nature of their contents. Another incurable form is the multilocular cyst, having numerous and non-communicating cells, the contents of which are almost always albuminous. The unilocular ovarian cysts, which are alone amenable to palliative and curative treatment, are fortunately those oftenest met with. M. Cruveilheir approves of leaving them alone as long as possible, for tapping once commenced has to be repeated with increasing frequency until the patient's strength becomes exhausted. He has had no experience in the employment of iodine, but he regards the facts now known as sufficient to justify its recommendation. The rock on which it may split is the morbid structure of the walls of the cyst, and the ease with which the accidental fibrous or fibro-serous tissue of which it is composed suppurates, or even becomes gangrenous. M. Cruveilhier cites cases in which such results have followed simple puncture, independently of the production of peritonitis. Still such cases are rare, and in his large practice he has only met with three cases of death following simple puncture,—the toleration of the operation seeming to increase with the frequency of its performance. In these cases, the cystic rather than the peritoneal inflammation which results, may yield to active treatment, while there are even instances of definitive cure having resulted, owing to adhesive inflammation having been excited by the puncture.

(To be continued.)

6. USE OF BELLADONNA IN ARRESTING SECRETIONS OF MILK IN SWOLLEN OR PAINFUL BREASTS. By *Samuel Willey*, M.D., of St. Paul.—"Upon reading Dr. Goolden's letter in the number of the *London Lancet* for October, 1856, I resolved to make early trial of the remedy, and five cases have been furnished me of tumefied and painful breasts, not differing in any essential feature from those reported by Dr. G., in which the free use of the extract of belladonna to the areola has acted like a specific in every instance. Once only did assistance seem required—citrate of magnesia having been given where protracted constipation obtained. Notes of the cases were kept without the remotest idea of publication, until I saw the article copied into the *Observer* for December. I hand you two extracts from my note-book.

"Mrs. B., aged 18, child aged 11 months, commenced menstruating regularly five months after birth of child; catamenia not occurring on 12th Nov., when expected, together with the appearance of an obstinate diarrhœa in the child, induced weaning, which was soon followed by distended and painful breasts.

Ordered, that the breasts should be well drawn, and an ointment, composed of ext. belladonna and simple cerate, two-thirds of the former to one of the latter, to be rubbed upon the nipples and areola every two hours. Three hours subsequently, found breasts flaccid and free from pain, much to the wonder of the lady, and the surprise of myself. No re-filling.

"Mrs. P., aged 21, having lost second child aged seven months, suddenly suffered with red, turgid, and throbbing breasts; pain much aggravated by riding to the cemetery, two miles distant, temperature 12° F. below zero. The application of belladonna *to the entire breasts* was made in this case, and complete relief was obtained in six hours. Extreme dilatation of the pupils with strabismus was produced, which passed away soon after the administration of paregoric in hot brandy sling. This was the only case where other than the nipples and areola were anointed."—*Cin. Med. Observer*, April, 1857.

